# Pain assessment tools in adults with communication disorders: systematic review and meta-analysis

**DOI:** 10.1186/s12883-024-03539-w

**Published:** 2024-02-17

**Authors:** Álvaro Sabater-Gárriz, Jesús Molina-Mula, Pedro Montoya, Inmaculada Riquelme

**Affiliations:** 1 Balearic ASPACE Foundation, Marratxí, Spain; 2 Department of Nursing and Physiotherapy, University of Balearic Islands, Palma, 07122 Spain; 3 Research Institute on Health Sciences (IUNICS), University of the Balearic Islands, Palma, 07122 Spain; 4 Health Research Institute of the Balearic Islands (IdISBa), Palma, 07010 Spain

**Keywords:** Communication Disorders, Nonverbal Communication, Observational Scales, Pain, Pain Assessment, Physiological Monitoring

## Abstract

**Background:**

Verbal communication is the "gold standard" for assessing pain. Consequently, individuals with communication disorders are particularly vulnerable to incomplete pain management. This review aims at identifying the current pain assessment instruments for adult patients with communication disorders.

**Methods:**

A systematic review with meta-analysis was conducted on PubMed, PEDRO, EBSCOhost, VHL and Cochrane databases from 2011 to 2023 using MeSH terms “pain assessment, “nonverbal communication” and “communication disorders” in conjunction with additional inclusion criteria: studies limited to humans, interventions involving adult patients, and empirical investigations.

**Results:**

Fifty articles were included in the review. Seven studies report sufficient data to perform the meta-analysis. Observational scales are the most common instruments to evaluate pain in individuals with communication disorders followed by physiological measures and facial recognition systems. While most pain assessments rely on observational scales, current evidence does not strongly endorse one scale over others for clinical practice. However, specific observational scales appear to be particularly suitable for identifying pain during certain potentially painful procedures, such as suctioning and mobilization, in these populations. Additionally, specific observational scales appear to be well-suited for certain conditions, such as mechanically ventilated patients.

**Conclusions:**

While observational scales dominate pain assessment, no universal tool exists for adults with communication disorders. Specific scales exhibit promise for distinct populations, yet the diverse landscape of tools hampers a one-size-fits-all solution. Crucially, further high-quality research, offering quantitative data like reliability findings, is needed to identify optimal tools for various contexts. Clinicians should be informed to select tools judiciously, recognizing the nuanced appropriateness of each in diverse clinical situations.

**Trial registration:**

This systematic review is registered in PROSPERO (International prospective register of systematic reviews) with the ID: CRD42022323655.

**Supplementary Information:**

The online version contains supplementary material available at 10.1186/s12883-024-03539-w.

## Introduction

Verbal communication is regarded as the "gold standard" for pain assessment [1], which is necessary for optimal management [2]. Since pain can be challenging to recognize by professionals, who frequently assess it based on their clinical impression, people with difficulties in verbal communication are particularly vulnerable to reduced or incomplete pain management [3–6]. Communication disorders affect people of all ages, although the prevalence and complexity of these conditions increase with age [7]. Thus, pain in people with communication difficulties due to dementia, intellectual disabilities or neurological conditions has been classically underestimated and, therefore, poorly treated [8, 9]. Moreover, many hospitalized people also experience temporary limitations in ability to communicate in situations such as recovering from anesthesia or being intubated [10].

Under-treated pain can result in both physical and psychological complications [11, 12]. However, evaluating pain in individuals with communication disorders is often viewed as a challenging and time-consuming task by healthcare professionals [13, 14]. Many of these professionals often report inadequate education and limited experience in dealing with patients in pain during their medical training, particularly in relation to vulnerable groups [14, 15]. Thus, a reliable and validated technique for pain evaluation in patients who are unable to self-report is urgently needed [16].

A multitude of observational tools is available to assess pain in this population, but there is not a clear consensus about the one to choose [11, 17]. Furthermore, these solutions are often considered to provide subjective, observer-dependent data [18–20], and some of these are only valid for a specific group of patients and context of care [21]. One way or another, there is an open debate about the usefulness of the non-verbal behaviors considered in these tools, as many of them can be non-specific or non-pain sensitive [17] or may determine secondary physiological indicators [18]. In order to address these issues, the clinical community is beginning to measure physiological signs that potentially can reflect pain, such as heart rate changes and heart rate variability, skin conductance and perfusion, changes in oxygen saturation, brain activity, pupil reactivity to light and expression of salivary metabolites, to cite a few [18, 22–26]. However, it needs to be pointed out that many of them are considered to lack sensitivity and specificity and cannot be used independently [27].

Taking all this into account, and due to the lack of evidence-based guidelines for pain assessment in the adult population [28], the main objective of this systematic review was to identify the different pain assessment methods currently used in adult patients with either permanent or temporary inability to communicate in any way. Specifically, we aimed at mapping and categorize existing instruments to evaluate pain in people with communication problems from which to commission primary research. Furthermore, the assessment of pain in people with communication problems was carried out through three constructs: pressure pain, suctioning pain and mobilization pain. These constructs could be included in the meta-analysis because they contained pre and post results or two comparison groups.

## Material and methods

### Design

A systematic mapping review with meta-analysis of pain assessment instruments in adult patients (≥18 years old) with communication disorders was performed. The PRISMA international standards were followed, as well as the Cochrane recommendations. This systematic review is registered in PROSPERO (International prospective register of systematic reviews) with the ID CRD42022323655.

### Search strategy

The bibliographic search was conducted from January 2021 to August 2023 in the following databases: Pubmed, PEDRO, Virtual Health Library (VHL), Cochrane and EBSCOhost (includes the following databases: CINAHL®Complet, Psychology & Behavioral Sciences Collection, Academic Search Complete, APA PsycInfo, Abstracts in Social Gerontology, MLA International Bibliography, APA PsycArticles and E-Journals. The search formulation was based on DeCS/MeSH Descriptors and free terms using Boolean operators and, in some cases, truncation to obtain the maximum number of compatible results and prevent loss of information. The Boolean combination was: (Pain assessment) AND (communication disorder OR non verbal communication).

According to Price's Law and Cochrane recommendations, the search was limited to results in the English language, interventions involving adult patients, and a publication period from 2011 to 2021. A secondary review was conducted in August 2023, encompassing publications from 2021 to 2023 to identify any additional clinical trials published during the analysis period. Additionally, some of the previously used terms were recognized and utilized as MeSH terms by the PubMed search engine: pain, pain assessment, communication disorders, nonverbal communication. Finally, a targeted snowball search strategy was implemented to include relevant studies that, due to the chosen publication period or other criteria, did not initially align with the search strategy but still provided valuable information related to the review's objectives. All identified studies were imported into the Mendeley bibliographic manager (Elsevier, London, England) with the intention of removing any duplicate entries.

### Selection criteria

The following inclusion criteria were followed in this systematic review: a) studies limited to humans; b) studies limited to patients over 18 years of age; c) studies limited to patients with an inability to self-report d) studies with control group or pre- and post- measurements that analyze or propose an assessment system that evaluates any behavioral (identifiable through observation) or physiological (identifiable through the measurement of any physiological parameter) responses related to a painful stimulus.

The exclusion criteria were a) No inability to self-report; b) No pain assessment models; c) Infant or neonate patients; d) Opinion pieces; e) Letters to the editor; f) Descriptive study protocols; g) Linguistic validations.

### Data collection

Two researchers (AS-G and IR) independently performed the selection and critical reading. In case of disagreement, a third investigator (JM) was consulted.

The selection of articles proceeded through four phases:

1. Identification: This phase involved searching different databases with subsequent elimination of duplicates.
2. Screening: Articles were evaluated based on their titles.
3. Selection: The eligibility of articles was assessed based on abstracts.
4. Inclusion: Potentially eligible studies were selected based on a critical reading of the full text.

The results were compiled in an Excel datasheet that included: title, author/s, year of publication, country of publication, financing, article source, study design, recruitment, sample (with demographic and clinical data), follow-up, measures, interventions, risk of bias, conclusions, and limitations.

Finally, an Excel table was created to categorize the analytical papers for assessing the feasibility of the meta-analysis (MA). The analytical coding table included the following variables: study code, title, year, author, assessment instrument, construct, pre-measurement (mean and SD), post-measurement (mean and SD), and sample size. In instances where complete data for the pre-post measurements were not available, requests were made to the authors (*n*=5).

### Assessment of risk of bias

The risks of bias of each study were assessed using the Cochrane Collaboration *Tool* as guidance [29]. This tool evaluates bias across seven specific domains: random sequence generation (selection bias), allocation concealment (selection bias), blinding of participants and personnel (performance bias), blinding of outcome assessment (detection bias), incomplete outcome data (attrition bias), selective reporting (reporting bias), and other bias. Each domain was categorized as "low risk," "high risk," or "moderate or uncertain risk." The overall risk was determined by weighing the risks observed in the various studies.

### Analysis and synthesis

#### Qualitative synthesis

A qualitative analysis was conducted to assess the strength of the relationship between the variables and various pain assessment methods described in patients with communication disorders. This analysis allowed us to filter and interpret the data for the meta-analysis. Some studies were not included in the meta-analysis due to the heterogeneity of the data or the absence of relevant outcome measures. The methodological quality of all seven studies included in the meta-analysis was assessed using the Critical Appraisal Skills Program tool, Spanish version (CASPe) [30]. Studies that achieved a score of 7 or higher were considered of sufficient quality for inclusion in both the review and meta-analysis. Each study's level of evidence, as determined by the CASPe score, was further categorized by the Scottish Intercollegiate Guidelines Network (SIGN) [31], along with its corresponding degree of recommendation.

We also provide the reliability findings from the studies, reporting measures such as Cronbach's alpha, kappa, or ICC. In the case of ICC, the interpretations are as follows: ICC < 0.5 = poor reliability, ICC 0.5-0.75 = moderate reliability, ICC 0.75-0.9 = good reliability, ICC > 0.90 = excellent reliability [32].

### Quantitative synthesis

When two or more outcome measures evaluated the same construct using similar instruments, the study was eligible for inclusion in a meta-analysis. The 'Meta-Essentials' Excel tool was used to conduct the meta-analysis [33]. Effect sizes were calculated by extracting pre-post sample sizes, means, and standard deviations (SD) from the selected studies. This was achieved by using the effect size or magnitude of the results, acknowledging the limitation that sometimes, even if the studies used the same construct, they might use similar but not identical scales. Dividing by a standard deviation allows studies that have applied different scales to measure the same construct or variable (e.g., measurement of pain) to express their results in a common metric (standard units). The quantification of results in a common metric is an essential requirement for applying subsequent statistical analysis techniques. Given the considerable diversity of scales and instruments used to measure the same variable in the phenomenon under study, the use of the standardized mean difference addresses the problem of heterogeneity in measurement instruments, enabling the statistical synthesis of the meta-analysis [34–36].

Despite the potential risk of introducing significant variability (heterogeneity), this approach was employed in an exploratory manner to offer additional insight into the overall landscape of current primary research and the prevailing state of measures used to assess pain in individuals with communication problems.

For continuous data, standardized mean differences (SMD) and 95% confidence intervals (CI) were calculated by dividing the mean of pre- and post- groups by the pooled SD. The SMD of the means proposed by Cohen in each study were weighted by the inverse of their variance to obtain the pooled index of the magnitude of the effect. Due to the heterogeneous nature of the selected studies, a random effects model was used. Finally, heterogeneity was evaluated using the inferential Q test proposed by Cochran, Pq test, Tau (T) square Tau ${T}^{2}$ and the ${I}^{2}$ hetero-geneity index with 95% CI. Heterogeneity was considered high or considerable when ${I}^{2}$ was >75% [37].

The asymmetries in the distribution of effect sizes, potentially resulting from publication bias or other forms of bias, were examined using two different approaches: Begg's strategy and Egger's test.

A sensitivity analysis was performed to test the influence of possible outliers and visualize the trends in the results. The thresholds for the interpretation of effect sizes were as follows: 0.1, small; 0.3, moderate; 0.5, large;0.7, very large; and 0.9, extremely large [33]. *P* < 0.05 was considered to indicate statistical significance.

It is important to note that for those studies that could not be incorporated into the meta-analysis due to either insufficient data or the utilization of different assessment instruments, solely a qualitative analysis was conducted (*n*=38).

## Results

### Search results

**Fig. 1:**
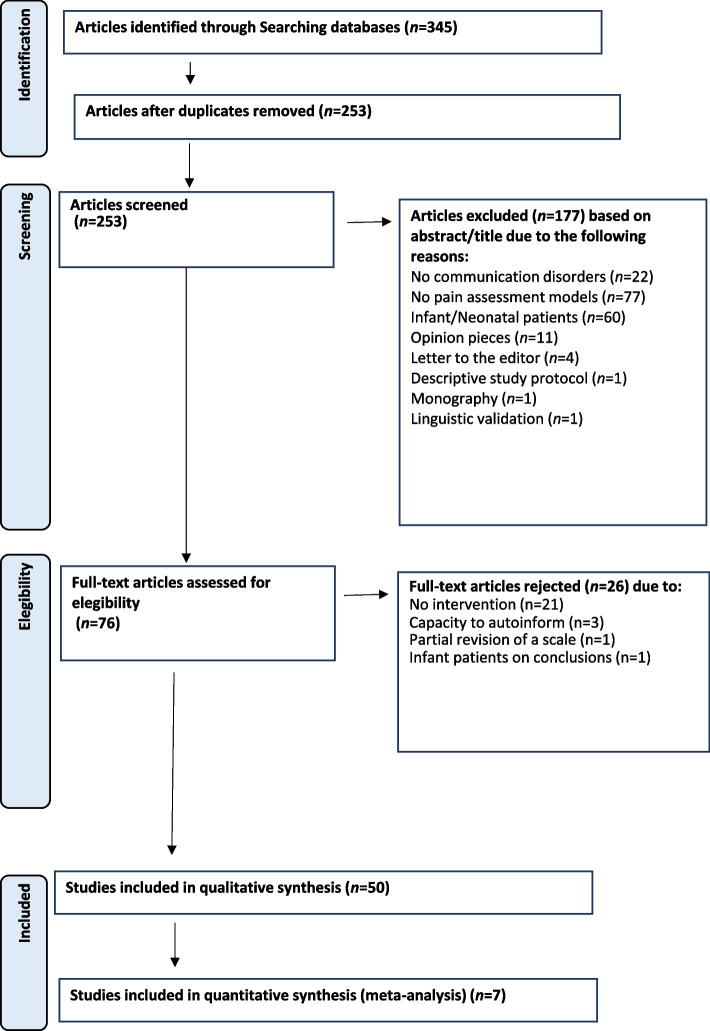
Flowchart. Selection process

The comprehensive search was completed in August 2023, yielding a total of 345 studies, of which 253 remained after eliminating duplicates. Once the eligibility criteria were applied and the abstracts were reviewed, the number of studies was reduced to 76 for subsequent full-text reading. Finally, 50 studies were included in the systematic review. Among them, twenty-two (44%) were clinical trials and were further examined to determine if they were suitable for inclusion in a meta-analysis. The distribution of the remaining studies was as follows: *n*=12 (24%) observational/descriptive; *n*=8 (16%) systematic reviews; *n*=5 (10%) linguistic validation; *n*=1 (2%) psychometric validation; *n*=1 (2%) secondary data analysis; *n*=1 (2%) scale validation. Ultimately, 8 studies provided enough data to perform the meta-analysis. Figure 1 illustrates the flowchart of the review based on the PRISMA criteria [38].

#### Description of included studies

The sample of participants in the included studies consisted of 1,054,982 individuals. The mean age of the participants was 63.71 years (SD= 12.20). Among the 50 selected studies, it should be noted that only 2 had the presence of a control group (*n*=45), referring to a group of individuals who were assessed without receiving a painful stimuli/procedure. Table S1 (see Supplementary material, table S1) presents the main characteristics of the selected studies.

Of all the studies, 36% (*n*=18) were conducted in North America, 34% (*n*=17) were conducted in Europe, 16% (*n*=8) were conducted in Asia, 6% (*n*=3) were performed in Oceania and 2% (*n*=1) in South America. Regarding the development of the studies, 28% (*n*=14) were multicentric.

Regarding the pain assessment systems used in the studies included in this review, the vast majority used observational scales 80% (*n*=40). Besides, the 4% (*n*=2) of the studies used computerized facial recognition technologies (Electronic Pain Assessment Tool -ePAT) and 16% (*n*=8) employed the evaluation of different physiological parameters such as brain activity, cardiac activity, muscle activity, respiratory activity, sweating or conductance of the skin. Tables S2, S3 and S4 (see Supplementary material, tables S2-S4) provide detailed information about the different systems used to assess pain.

Classifying the studies by the characteristics of the patients, 29.54% (*n*=13) focused on elderly patients with dementia, 22.73% (*n*=10) on patients with mechanical ventilation, 12% (*n*=6) on patients with brain damage, 8% (*n*=4) on patients with cerebral palsy, 6% (*n*=3) on elderly patients with communication disorders,6% (*n*=3) on patients with intellectual disability, 6% (*n*=3) on critical patients, 6% (*n*=3) on patients with aphasia post-stroke, 2% (*n*=1) on cancer patients, 2% (*n*=1) on patients with acute pain, and 2% (*n*=1) on patients in a vegetative state/minimal consciousness.

Regarding the painful procedure assessed (factor), the review showed great heterogeneity. Most of the studies [24% (*n*=12)], assessed pain produced by mobilization or transfer of patients or by tracheal aspiration [20% (*n*=10)]. Further, the 16% (*n* =8) of the studies assessed pain due to a routine assessment, 16% (*n*=8) due to routine activities, 10% (*n*=5) due to painful pressure (pain produced by direct pressure on the skin with a pressure algometer) or by puncture 6% (*n*=3) (2 injections, 1 puncture with neuropen), 4% (*n*=2) due to movement (nonspecific), and 4% (*n*=2) due to walking among others.

#### Reliability findings

Out of all the selected studies utilizing observational scales, a total of 27 studies (67.5%) reported reliability results (detailed in the Supplementary material, table S5). Given the diversity in the types of scales employed across these studies, as well as the variations in the populations under assessment and the methods of reliability evaluation, we have categorized the studies to facilitate the synthesis and comparative analysis of their results (Table 1).

**Table 1:** Synthesis of findings for interrater reliability, test-retest reliability and internal consistency of pain observational scales

**Scale**	**Population**	**Interrater Reliability**^**b**^
PAINAD	Cancer patients	0.97-0.98 (ICC); k=80
PACS LAC	Elderly patients with dementia	0.917 (ICC 2,1)
CPOT	Critical patients	86-100%
FACS	Intellectual disabled patients	93%
CPOT	Mechanically ventilated patients	k=84
		**Test-Retest Reliability**^**b**^
PACSLAC	Patients with post-stroke aphasia	0.88-0.95 (ICC)
FLACC	Elderly patients with dementia	0.73 (ICC)
		**Internal Consistency**^**b**^
CPOT	Mechanically ventilated patients	0.95 (Cronbach α)
Pain Indicators for Brain-Injured Patients^a^	Patients with brain injury	0.95 (Cronbach α)
COMFORT	Mechanically ventilated patients	0.90 (Cronbach α)
PACSLAC II	Patients with post-stroke aphasia	0.83 (Cronbach α)
BPS	Mechanically ventilated patients	0.80-0.94 (Cronbach α) 0.77-0.95 (ICC)
PAINAD	Critical patients	0.80
PACSLAC	Patients with post-stroke aphasia	0.71 (Cronbach α)
NOPPAIN	Elderly patients with dementia	0.80-0.97 (Cronbach α)
PAINAD	Cancer patients Patients with brain injury	0.72-0.75 (Cronbach α)

^a^Items selected from CPOT and BPS

^b^*ICC* intraclass correlation coefficient (different types added when reported), *ICC 2,1* two-way random absolute agreement, *ICC<0.5* poor reliability, *ICC 0.5-0.75* moderate reliability, *ICC 0.75- 0.9* good reliability, *ICC > 0.90* excellent reliability [39]

The methodological quality of all the 7 studies included in the meta-analysis according to CASPe and SIGN, is specified in table S6 (see Supplementary material, table S6 ).

#### Quantitative analysis

Three meta-analyses were performed among 7 studies. Specifically, variables such as Pressure pain, (pain produced by a direct pressure on the skin with a pressure algometer) (Table 2), Suctioning Pain (pain produced by a tracheal suctioning) (Table 3) and Mobilization Pain (pain produced by a postural change or transference) (Table 4) were analyzed quantitatively. Pressure pain was assessed with PCSLACII and NCS (Nociception Coma Scale); Suctioning pain was assessed with ESCID, BPS and CPOT; and Mobilization pain was assessed with ESCID and BPS.

**Table 2:**
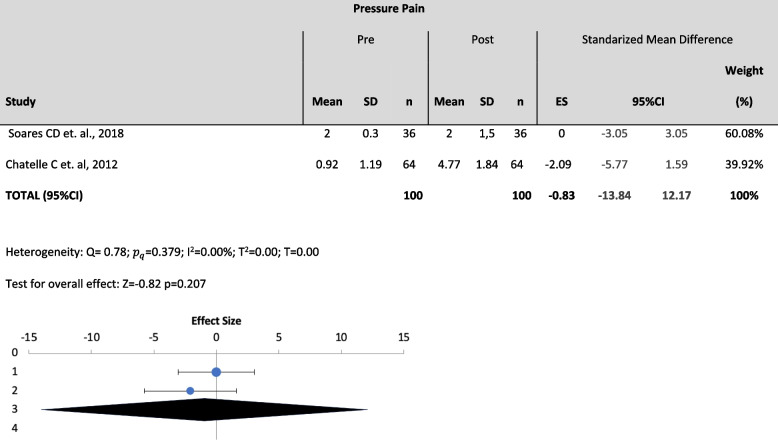
Meta-analysis forest plot for pressure pain

**Table 3:**
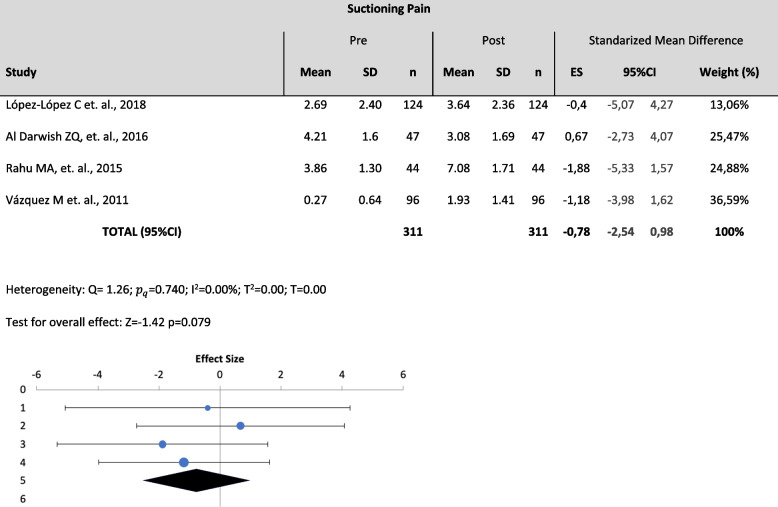
Meta-analysis forest plot for suctioning pain

**Table 4:**
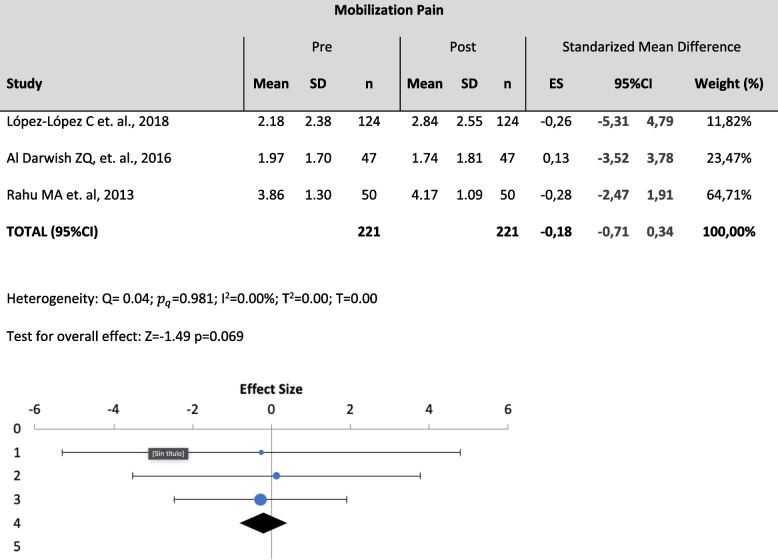
Meta-analysis forest plot for mobilization pain

The effect size has been moderate or large in the studies included in the meta-analysis. We highlight the effect size of López-López C et. al., 2018 of -0.4 (moderate) and of Al Darwish ZQ, et. al., 2016 of 0.67 (large). The rest has a small effect between 0.1 and 0.3

​The effect size findings reveal that a large number of comparisons fall into the small and moderate magnitude category, which can generate errors in the interpretation of the results based on the p value of the different studies and therefore, take your conclusions with caution.

The results showed low heterogeneity in all the analyzed variables (${I}^{2}$= 0% for all variables) and there were no statistically significant changes on the outcomes of the different assessment tools between pre-pain and post-pain assessments [(*p* >0.05); Pressure (${I}^{2}$= 0%; Z=0.82; *p*=0.207), suctioning (${I}^{2}$= 0%; Z=-1.42; *p*=0.079), mobilization (${I}^{2}$= 0%; Z=-1.49 *p*=0.069)]. Thus, despite the to the lack of significance and the absence of heterogeneity, the meta-analysis cannot conclude the usefulness of any of the scales under study to statistically differentiate pre- and-post pain using the cited variables.

#### Risk of bias assessment

**Fig. 2:**
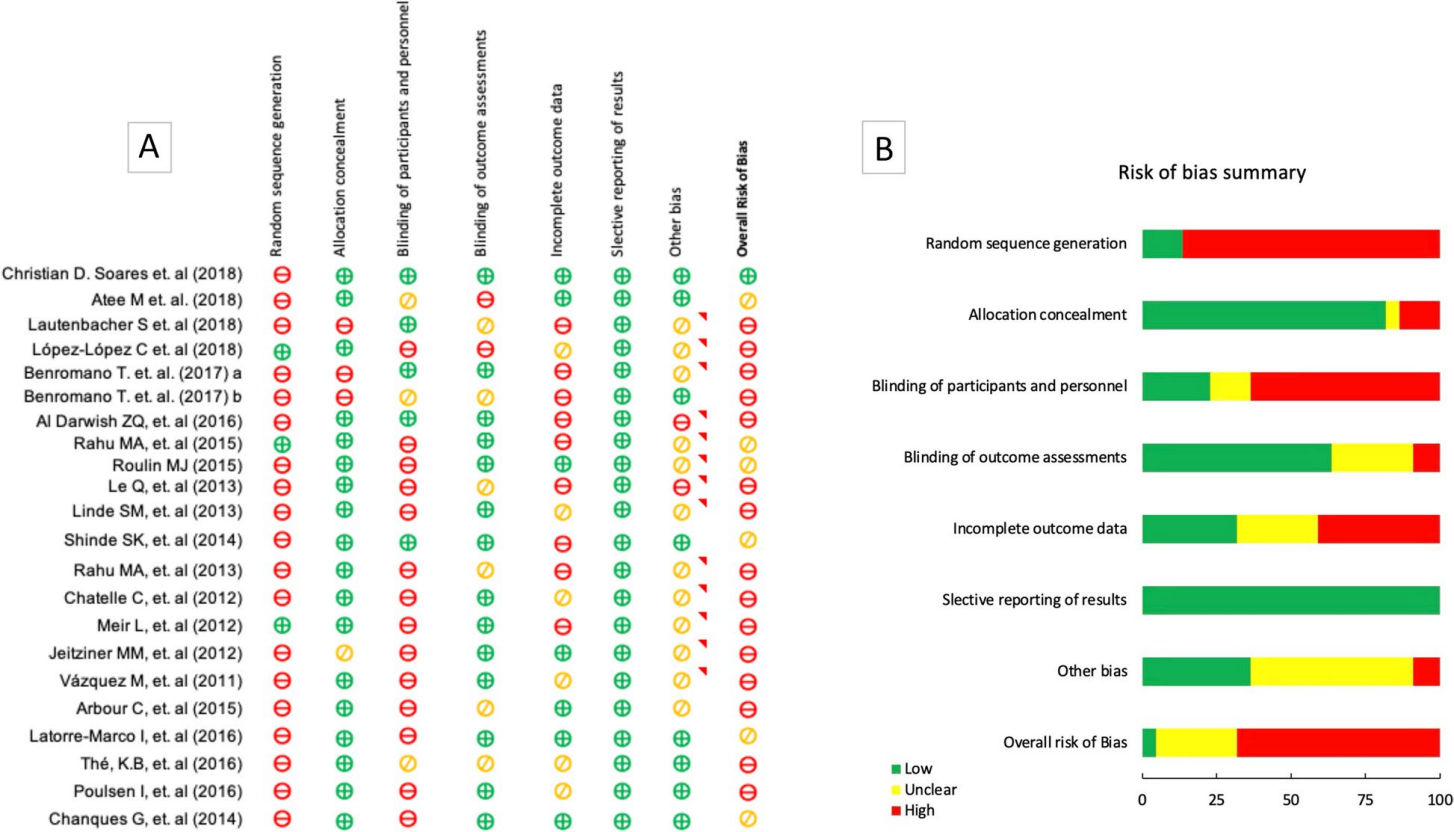
Risk of bias assessment: Overall risk of bias **A** Risk of bias summary **B**

High risk of bias was found in 15 studies: Lautenbacher et al [40], López-López et al [41], Benromano et al (a&b) [24, 25], Al Darwish et al [26], Le et al [42], Linde et al [43], Rahu et al [44], Chatelle et al [45], Meir et al [46], Jeitziner et al [47], Vázquez et al [48], Arbour et al [49], Thé et al [50] and Poulsen et al [51] ; unclear risk of bias was found in 6 studies: Atee et al [52], Rahu et al [53], Roulin et al [54], Shinde et al [55], Latorre-Marco et al [56] and Chanques et al [57]. Only one study had a low risk of bias (Soares et al., 2018) [58]. According to the ROBINS-I tool (Risk Of Bias tool to assess Non-randomized Studies of Interventions), the areas that were most likely to increase the risk of bias were random sequence generation and blinding of participants and personnel, while bias due to selective reporting of result as had the lowest risk (Fig. 2).

## Discussion

Our review revealed a wide array of pain assessment tools designed for non-communicative patients, ranging from physiological variables to observational scales. Among these tools, observational scales are the most commonly employed instruments for evaluating pain in individuals with communication disorders. The diversity of methods poses a challenge in designating a single scale as the gold standard for pain assessment in adults with communication disorders. Nevertheless, specific observational scales appear to be particularly suitable for identifying pain during certain potentially painful procedures, such as suctioning and mobilization, in these populations. Additionally, specific observational scales appear to be well-suited for particular conditions, notably in the case of mechanically ventilated patients.

Evidence underscores the importance of using observational tools since relying solely on self-reports is inadequate for assessing pain in patients with communicative disorders [59]. Our study revealed a wide variety of studies employing different scales, often with small sample sizes and a high risk of bias. This diversity hinders a comprehensive and reliable analysis, resulting in a low level of confidence according to this systematic review and analytical study. Indeed, the meta-analysis showed low results when examining pain changes before and after three painful procedures.

Nonetheless, our meta-analyses identified consistent trends in the effectiveness of specific scales used in pain assessments during certain procedures, such as mobilization and aspiration. These procedures should be monitored for pain in these vulnerable populations. While these findings may not be universally applicable, they do suggest promising avenues for further research.

Other tools that employ a combination of specific facial codes and common pain behaviors [60] have demonstrated favorable reliability properties [61]. Nonetheless, to the best of our knowledge, there are no studies concerning the correlation of their scores with those obtained from other assessment tools. In addition, this systematic review has unveiled a range of physiological measures, reflecting efforts to utilize objective markers for pain evaluation. However, even in environments with readily available access to these instruments, such as ICUs, the use of observational scales remains more prevalent [13, 62–64]. While this review did not yield sufficient data to assess their reliability properties, these measures may emerge as an alternative or complement to behavioral scales. They warrant further consideration in future studies to ensure a multidimensional approach to pain assessment [27].

This review has several limitations. The use of effect size in similar but not identical instruments introduces an important element of variability in the meta-analysis that can compromise heterogeneity even if analyzing the same construct. This is not an exclusive difficulty of meta-analysis, since the wide variety of characteristics inherent to the study subjects makes it necessary to design a uniform protocol, carry out a rigorous process of subject selection and perform a careful analysis of the influence on the results of extreme cases. Moreover, this aspect has been seen in previous systematic reviews, which also concluded that no single scale could be universally recommended [65, 66]. Furthermore, not being able to report all the confidence intervals before the absence of data provided by the authors, of the included studies, represents a reproducibility bias of the meta-analysis. This means that it is not possible to fully determine the impact of the findings.

In conclusion, the predominant method of pain assessment in adults with communication disorders involves the use of observational scales, with certain scales demonstrating promising psychometric properties for specific populations. Nevertheless, the existing diversity in assessment tools and study designs prevents the selection of a universally suitable scale for evaluating pain across all adults with communication disorders.

Current evidence does not strongly favor one scale over others for clinical practice. To enhance their recommendation in clinical guidelines, further research with more rigorous study designs is imperative. In this regard, we acknowledge the existence of at least two major groups [67, 68] that are conducting psychometric tests on items from various observational scales and analyzing those items that best predict clinicians' evaluations of pain intensity, in order to provide tools with high reliability and validity, such as the Pain Intensity Measure for Persons with Dementia and the Pain Assessment in Impaired Cognition (PAIC-15 scale).

It is advisable to carry out studies of diagnostic accuracy (STARD) and prognosis (REMARK) to, based on this review, establish the instruments that offer the most sensitivity and specificity.

Moreover, there is a need for exploration of alternative instruments that can complement the information provided by behavioral scales, including facial recognition systems or physiological signals. Such exploration can help mitigate the observer-dependent, subjective nature of observational assessment systems.

### Supplementary Information

**Additional file 1:** **Table S1.** Description of selected studies [69–84]. **Table S2.** Population, pain observational scales and painful procedure of the included studies. **Table S3.** Facial recognition measures, population and painful procedure of the included studies. **Table S4.** Physiological measures, population and painful procedure of the included studies. **Table S5.** Reliability findings of pain observational scales included in the systematic review. **Table S6.** Methodological quality of the studies included in the meta-analysis.

## Data Availability

The datasets generated and/or analysed during the current study are available from the corresponding author on reasonable request.
