# Supplementary material for: Pain assessment tools in adults with communication disorders: systematic review and meta-analysis
Source: BMC Neurol. 2024 Feb 17;24:66. doi: 10.1186/s12883-024-03539-w (PMC10873938; doi:10.1186/s12883-024-03539-w)
Supplement: Supplementary file 1 — Additional file 1: Table S1. Description of selected studies [69–84]. Table S2. Population, pain observational scales and painful procedure of the included studies. Table S3. Facial recognition measures, population and painful procedure of the included studies. Table S4. Physiological measures, population and painful procedure of the included studies. Table S5. Reliability findings of pain observational scales included in the systematic review. Table S6. Methodological quality of the studies included in the meta-analysis. [file 12883_2024_3539_MOESM1_ESM.pdf]

SUPPLEMENTARY MATERIAL

Table S1. Description of selected studies.

Author	Year of publication	Data collection	Country	Intervention (n)	Controls	Sex				Average Age	
						Male		Female			
						n	(%)	n	(%)	Mean	SD
de Vries, et al.[69]	2023	2014-2018	Netherlands	60		32	57%	28	43%	79.3	8
Haghi M, et al.[70]	2020	2017	Iran	138		64	46,37%	74	53,62%	74,5	8,9
Morrison, R. A, et. al. [71]	2020	2010-16	USA	1,036,806		411612	39,80%	625194	60,20%	N/A	N/A
Fox MA et al. [1]	2019	2014-15	USA	48		13	27%	35	73%	29,2	13
Muñoz-Narbona, L, et. al.[72]	2019	2017	Spain	325		189	58,20%	136	41,80%	71,1	13,4
Soares CD. et. al., [57]	2018		USA	36		19	52,70%	17	47,30%	61	N/A
Atee M et. al. [59]	2018	2016	Australia	10		5	50%	5	50%	74,4	5,9
Lautenbacher S et. al. [39]	2018		Germany	42		22	52,40%	20	47,60%	76,7	7,3
López-López C et. al. [40]	2018	2014-15	Spain	124		96	77,40%	28	22,60%	45,9	16,4
Riquelme I et. al.[73]	2018		Spain	96		63	65,60%	33	34,40%	18,9	12,7
Pickering G et. al.[74]	2018		Spain, Portugal, Italy, Australia, Turkey	176		60	34,10%	116	65,90%	83,4	7,9
Moon Y et. al. [75]	2017		S.Korea	98		38	31,8	60	68,20%	77,3	6,3
Atee M. et. al. [51]	2017	2015-16	Australia	40		11	30%	29	70%	79,7	9,1
Benromano T. et. al. [24]	2017		Israel	16	25	8	50%	8	50%	36,4	7,3
Benromano T et. al.[25]	2017		Israel	13	20	6	46,20%	7	54,80%	34,5	4,9

Al Darwish ZQ, et. al.[26]	2016	2014	Saudi Arabia	47		27	57%	20	43%	55,5	20,2
Gregersen, M et al. [76]	2016		Denmark	50		20	40%	30	60%	85,4	7,9
Latorre-Marco, I., et. al [55]	2016		Spain	190		135	71%	55	29%	61	N/A
Thé, K.B, et. al [49]	2016		Brazil	50		11	22%	39	78%	87,8	6,5
Poulsen, I., et. al., [50]	2016		Denmark	26		16	61,50%	10	48,50%	56,5	N/A
Rahu MA, et. al. [52]	2015		USA	150		78	52%	72	48%	53	15,5
Ford B, et. al. [77]	2015		USA	78		N/A	N/A	N/A	N/A	N/A	N/A
Roulin MJ, et. al. [53]	2015		Switzerland	116		66	56,90%	50	43,10%	56,2	1,6
Arbour, C., et. al. [48]	2015		Canada	25		17	68%	8	32%	56,5	N/A
Apinis C, et. al. [78]	2014		Canada	59		30	50,84%	29	49,16%	82	11,7
Chan S, et. al. [79]	2014		Canada	124		88	71%	36	29%	83,94	7,95
Shinde SK,et. al., [54]	2014		USA	44		23	52%	21	48%	46	9,87
Neville, C, et. al. [80]	2014		Australia	126		22	17%	104	83%	85,2	6,6
Chanques, G, et. al. [56]	2014	2012	USA	30		11	36,70%	19	63,30%	67	N/A
Le Q, et. al. [81]	2013		Canada	9		9	100%	0	0%	25.5	58
Linde SM, et. al. [42]	2013		USA	30		23	77%	7	23%	70,43	9,63
Rahu MA, et. al. [41]	2013		USA	50		26	52%	24	48%	52,5	17,2
Takai Y, et. al [82]	2013	2012	Japan	117		30	25,60%	87	74,40%	82,3	9,5
Pickering, et. al [83]	2013		France	33		19	57,57%	14	42,42%	38	3
Chatelle C,et. al [44]	2012		Belgium	64		40	62,50%	24	37,50%	N/A	N/A
Meir L, et. al [45]	2012		Isrrael	228		137	60%	91	40%	38,7	N/A
Jeitziner MM, et. al. [46]	2012		Switzerland	21		9	42,80%	12	57,20%	70	N/A

Paulson-Conger M, et. al. [84]	2011		USA	100		60	60%	40	40%	55,52	17,5
Vázquez M, et. al., [47]	2011	2007-8	Spain	96		55	57,30%	41	42,70%	62,11	14,38
Sheu E, et. al. [85]	2011		Canada	60		11	18,30%	49	81,40%	84	N/A
Gélinas, C., et. al. [86]	2011		Canada	90		57	63,30%	33	36,70%	59,8	18,94

N/A: not available; SD: standard deviation

Table S2. Population, pain observational scales and painful procedure of the included studies.

.

Population	Author	Year of publication	Scale	Painful procedure	n	Control
Elderly with dementia	Haghi M, et al.	2020	*PACSALC-II*	Routine activities	138	
	Morrison, R. A, et. al.	2020	*MDS 3.0*	Routine assessment	1,036,806	
	Lautenbacher S et. al.	2018	*PAIC-FACE-SCALE*	Painful pressure	42	
	Moon Y et. al.	2017	*FLACC*	Routine assessment	98	
	Thé, K.B, et. al	2016	*PACSALC-II*	Exposure to painful circumstances	50	
	Ford B, et. al.	2015	*NOPPAIN*	Routine activities	78	
	Chan S, et. al.	2014	*PACSLAC*<br>*PACSLAC-II*	Injection, mobilization	124	
	Neville, C, et. al.	2014	*Doloplus-2*<br>*APS*<br>*CNPI*	Routine assessment	126	
	Takai Y, et. al	2013	*PACSLAC*	Mobilization, Walking	117	
	Sheu E, et. al.	2011	*PACSLAC*<br>*NOPPAIN*<br>*PAINAD*<br>*Doloplus-2*<br>*Mahoney Pain Scale*<br>*APS*	Routine activities, walking	60	
	Apinis C, et. al.	2014	*PACSLAC*<br>*PAINAD*	Mobilization	59	
Elderly with Communication Disorders	Apinis C, et. al.	2014	*PACSLAC*<br>*PAINAD*	Mobilization	59	

	Pickering G. et. al.	2018	*Algoplus*	Routine activities	176	
	Gregersen, M et al.	2016	*APS*	Movement	50	
Mechanically ventilated patients	López-López C et. al.	2018	*ESCID*	Suctioning/mobilization	124	
	Al Darwish ZQ, et. al.	2016	*BPS*<br>*CPOT*<br>*NVPS*	Suctioning/ mobilization	47	
	Latorre-Marco, I., et. al	2016	*ESCID*<br>*BPS*	Suctioning/ mobilization	190	
	Rahu MA, et. al.	2015	*FLACC*<br>*BPS*<br>*ANVPS*<br>*COMFORT*<br>*PABS*	Suctioning/ mobilization	150	
	Linde SM, et. al.	2013	*CPOT*	Mobilization	30	
	Rahu MA, et. al.	2013	*FLACC*<br>*BPS*<br>*ANVPS*<br>*COMFORT*<br>*PABS*	Suctioning	50	
	Jeitziner MM, et. al.	2012	*FACS*<br>*RASS*<br>*GCS*	Suctioning	21	
	Vázquez M, et. al	2011	*CPOT*	Suctioning	96	
Cerebral palsy	Fox MA et al.	2019	*FLACC*	Routine assessment	48	
	Riquelme I et. al.	2018	*NCCPC*	N/A	96	
	Benromano T et. al. b)	2017	*Pyramid Pain scale*<br>*FACS*	Painful pressure	13	20
Brain injury	Poulsen, I., et. al	2016	*"PAS Preliminar"*	Mobilization	26	
	Roulin MAcute pain, et. al.	2015	*BPS*<br>*CPOT*	Mobilization	116	
	Le Q, et. al.	2013	*PBAT*<br>*CPOT*	Mobilization	9	
	Muñoz-Narbona, L, et. al.	2019	*PAINAD*	Movement	325	
Intellectual disability	Shinde SK,et. al	2014	*PADS*<br>*FACS*	Prick with neuropen	44	
	Meir L, et. al	2012	*NCAPC*	Injection	228	

Critical patients	Chanques, G, et. al.	2014	*BPS*<br>*CPOT*<br>*NVPS*	Routine activities, Suctioning/mobilization	30
	Paulson-Conger M, et. al.	2011	*PAINAD*<br>*CPOT*	Routine assessment	100
	Gélinas, C., et. al.	2011	*CPOT*	Routine assessment	90
Acute pain	Pickering, et. al	2013	*Algoplus*	Routine assessment	33
Vegetative/minimum consciousness	Chatelle C,et. al	2012	*NCS*	Painful pressure	64
Cancer patients	Muñoz-Narbona, L, et. al.	2019	*PAINAD*	Movement	325
Post stroke aphasia	Soares CD. et. al	2018	*PACSALC-II*	Painful pressure	36

Abbreviations:
PACSALC: Pain Assessment Checklist for Seniors with Limited Ability to Communicate
PACSALC-II: Pain Assessment Checklist for Seniors with Limited Ability to Communicate Version II
FLACC: Face, Legs, Activity, Cry, Consolability
ESCID: Escala de Conductas Indicadoras de Dolor
NCCPC: Non-Communicating Children Pain Checklist
BPS: Behavioral Pain Scale
NRS: Numeric Rating Scale
ANVPS: Adult Non-Verbal Pain Scale
PABS: Pain Behavior Scale
NOPPAIN: Non-Communicative Patient's Pain Assessment Instrument
PBAT: Pain Behavior Assessment Tool
PADS: Pain and Distress Scale
FACS: Facial Action Coding System
NCS: The Nociception Coma Scale
PAINAD: Pain Assessment in Advanced Dementia
APS: The Abbey Pain Scale
MDS 3.0: Minimum Data Set 3.0
CNPI: Checklist of Nonverbal Pain Indicators
”PAS Preliminar”: Pain Assessment Scale "Preliminar"
PAIC-FACE-SCALE: Pain and Impaired Cognition-Face-Scale
CPOT: Critical Care Pain Observation Tool
NVPS: The Nonverbal Pain Scale
NCAPC: Non-Communicating Adults Pain Checklist
RASS: Richmond Agitation Sedation Scale
GCS: Glasgow Coma Scale

Table S3. Facial recognition measures, population and painful procedure of the included studies.

Author	Year of publication	Electronic pain assessment	Population	Painful procedure	n	Control
Atee M et. al.	2018	ePAT (electronic pain assessment tool)	A	Routine assessment	10	0
Atee M. et. al.	2017	ePAT (electronic pain assessment tool)	A	Routine activities	40	0

A: elderly with dementia.

Table S4. Physiological measures, population and painful procedure of the included studies.

Author	Year of publication	Physiological measure	Population	Painful procedure	n	Control
Benromano T. et. al. a)	2017	Brain activity (contact heat–evoked potentials)	H	Thermal stimulus	16	25
Benromano T et. al. b)	2017	HR, HRV, PA, GSR	C	Painful pressure	13	20
Arbour, C., et. al.	2015	Brain activity (Bispectral Index System)	D	Mobilization	25	
Jeitziner MM, et. al.	2012	BP, HR, SpO2, RR, TV, sweating, pupil size, body movement, muscle tone	F	Suctioning	21	
Vázquez M, et. al	2011	BP, HR, RR, SpO2, sweating	F	Suctioning	96	

HR: Heart rate; HRV: Heart rate variability; PA: Pulse amplitude; GSR: Galvanic skin response; BP: Blood pressure; SpO2: Arterial blood oxygen saturation; RR: Respiratory rate; TV: Tidal volume.
C: Cerebral Palsy; D: Brain injury; H: Intellectual disability; F: Mechanically ventilated patients

Table S5. Reliability findings of pain observational scales included in the systematic review

Scale	Research Study	Population	Reliability findings
**PACSLAC**			
	Takai Y, et. al	A	Interrater reliability: 0.917 (ICC 2,1)<br>Test-retest reliability: 0.600 (ICC 1, k)
	Sheu E, et. al.	A	Interrater reliability: r=0.25-0.53 (Pearson product-moment correlation coefficient)
	de Vries, et al.	B	Internal consistency: 0.71 Cronbach α<br>Test-retest reliability: 0.88-0.95(ICC- type not reported)
**PACSLAC II**			
	Haghi M, et al.	A	Interrater agreement: 0.76 Cronbach α
	Chan S, et. al.	A	Interrater reliability: k = 0.63
	Soares CD. et. al	B	Interrater reliability: 0.87-0.94 Cronbach α<br>Internal consistency: 0.827 Cronbach α
	Gazoni, F. M, et. al	A	Interrater reliability: 85.2% (ICC -type not reported)<br>Test-retest reliability: 64.3% (ICC- type not reported)<br>Reliability: k=0.381
**FLACC**			
	Fox MA et al.	C	Interrater reliability: 0.41-0.57 (ICC -type not reported)
	Moon Y et. al.	A	Test-retest reliability: 0.73 (ICC -type not reported)
**ESCID**			
	López-López C et. al.	F	Interrater reliability: k >0.84
	Latorre-Marco, I., et. al	F	Internal consistency: 0.85 Cronbach α<br>No significant intrarater differences (p = 0.241)<br>Interrater reliability: difference of 0.0 or 0.1 points between the two observers
**Algoplus**			
	Pickering G. et. al.	G	Internal consistency: >0.6 (KR20s)**<br>Test-retest reproducibility: k=0.69 to 0.84<br>Interrater reliability: k=0.69 to 0.84
	Pickering, et. al	J	Internal consistency: 0.51 Cronbach alpha α
**BPS**			
	Al Darwish ZQ, et. al.	F	Internal consistency: 0.77-0.95 (ICC -type not reported)
	Latorre-Marco, I., et. al	F	Interrater reliability: "High" (difference of 0.0 or 0.1 points between the two observers)
	Chanques, G, et. al.		Interrater reliability: k=0.81<br>Internal consistency: 0.80 Cronbach α
	Rahu MA, et. al.	F	Test-retest reliability tested during rest and procedure: r = 0.71 at rest and r = 0.50 during procedure<br>Internal consistency: 0.94 Cronbach α
**CPOT**			
	Al Darwish ZQ, et. al.	F	Internal consistency: 0.95 Cronbach α<br>Interrater reliability: r=0.74-0.99; k=0.81
	Paulson-Conger M, et. al.	I	Internal consistency reliability: 0.72 Cronbach α
	Linde SM, et. al.	F	Interrater reliability: 0.87 (weighted Kappa)
	Vázquez M, et. al	F	Interrater reliability: k=0.79-1; 97-100%
	Gélinas, C., et. al.	I	Interrater reliability: 86-100%
**NVPS**			

Al Darwish ZQ, et. al.	F	Internal consistency: 0.86 Cronbach α
Chanques, G, et. al.	I	Interrater reliability: k=0.71<br>internal consistency: 0.76 Cronbach α
**ANVPS**		
Rahu MA, et. al.	F	Internal consistency: 0.78 Cronbach α
**COMFORT**		
Rahu MA, et. al.	F	Internal consistency: 0.90 Cronbach α
**NOPPAIN**		
Ford B, et. al.	A	Interrater reliability: r = 0.7 -1.0<br>Test-retest reliability: r = 0.68-0.95<br>Internal consistency: 0.80-0.97 Cronbach α
Sheu E, et. al.	A	Interrater reliability: k=0.23
**PADS**		
Shinde SK,et. al	H	Interrater reliability: r= 0.82
**FACS**		
Shinde SK,et. al	H	Interrater agreement: 93%
Benromano T et. al. b)	C	Interrater agreement: 0.67-0.92 (ICC -not reported)
**PAINAD**		
Muñoz-Narbona, L, et. al.	D,E	Internal consistency: 0.72-0.75 Cronbach α<br>Interrater reliability: k=>0.80 ; 0.97-0.98 (ICC -not reported)
Paulson-Conger M, et. al.	I	Internal consistency reliability: 0.80
Sheu E, et. al.	A	Interrater reliability: k=0.15-0.42
**APS**		
Sheu E, et. al.	A	Interrater reliability: k=0.07-0.31
Neville, C, et. al.	A	Test-retest reliability: 0.61-0.68 Cronbach α<br>Internal consistency: 0.65-0.79 Cronbach α<br>Interrater reliability: 0.70-0.75 (ICC -type not reported); 0,33-0.47 (weighted Kappa)
Gregersen, M et al.	G	Interrater reliability: 0.84 (ICC -not reported)<br>Internal consistency: 0.52 Cronbach α
**Mahoney Pain Scale**		
Sheu E, et. al.	A	Interrater reliability: k=0.30-0.32
**Doloplus-2**		
Sheu E, et. al.	A	Interrater reliability: k=0.04-0.38
Neville, C, et. al.	A	Test-retest reliability: 0.70 Cronbach α<br>Internal consistency: 0.85-0.86 Cronbach α<br>Interrater reliability: 0.73-0.81 (ICC -type not reported); 0,42-0.50 (weighted Kappa)
**CNPI**		
Neville, C, et. al.	A	Test-retest reliability: 0.44-0.56 Cronbach α<br>Internal consistency: 0.75-0.82 Cronbach α<br>Interrater reliability: 0.58-0.71 (ICC -type not reported); 0,43-0.53 (weighted Kappa)
**Pain Indicators for Brain-Injured Patients***		
Roulin MJ, et. al.	D	Internal consistency: 95% Confindence Interval

** Items selected from CPOT and BPS;** (KR20s): Kuder-Richardson's α coefficient ***ICC: intraclass correlation coefficient (different types added when reported); ICC 2,1: two-way random absolute agreement; ICC 1, k: One-way random, average measures.*

ICC<0.5 = poor reliability, ICC 0.5-0.75 = moderate reliability, ICC 0.75- 0.9 = good reliability, ICC > 0.90 = excellent reliability [80]
A: Elderly with dementia; B: Post stroke aphasia; C: Cerebral palsy; D: Brain injury; E: Cancer patients; F: Mechanically ventilated patients; G: Elderly with Communication Disorders; H: Intellectual disability; I: Critical patients; J: Acute pain; K: Vegetative/minimum consciousness

Table S6. Methodological quality of the studies included in the meta-analysis.

	**CASPE Score**	**SIGN Level of evidence**
Soares CD et. al.,, 2018	8	2+
Chatelle C et. al., 2012	6	2-
López-López C et. al., 2018	7	2-
Al Darwish ZQ, et. al., 2016	7	2-
Rahu MA, et. al., 2015	7	2+
Rahu MA et. al., 2013	6	2-
Vázquez M et.al, 2011	7	2-

CASPe: Critical Appraisal Skills Program tool Spanish (range 1-11). SIGN: Scottish Intercollegiate Guidelines Network (range 1++- 4)
